# Monitoring Natural SARS-CoV-2 Infection in Lions (*Panthera leo*) at the Barcelona Zoo: Viral Dynamics and Host Responses

**DOI:** 10.3390/v13091683

**Published:** 2021-08-25

**Authors:** Hugo Fernández-Bellon, Jordi Rodon, Leira Fernández-Bastit, Vanessa Almagro, Pilar Padilla-Solé, Cristina Lorca-Oró, Rosa Valle, Núria Roca, Santina Grazioli, Tiziana Trogu, Albert Bensaid, Jorge Carrillo, Nuria Izquierdo-Useros, Julià Blanco, Mariona Parera, Marc Noguera-Julián, Bonaventura Clotet, Ana Moreno, Joaquim Segalés, Júlia Vergara-Alert

**Affiliations:** 1 Parc Zoològic de Barcelona, Parc de la Ciutadella s/n, 08003 Barcelona, Spain; hfernandez@bsmsa.cat (H.F.-B.); valmagro@bsmsa.cat (V.A.); ppadilla@bsmsa.cat (P.P.-S.); 2 Institut de Recerca i Tecnologia Agraroalimentàries (IRTA), Centre de Recerca en Sanitat Animal (CReSA, IRTA-UAB), Campus de la UAB, 08193 Cerdanyola del Vallès, Spain; jordi.rodon@irta.cat (J.R.); leirapaula.fernandez@irta.cat (L.F.-B.); cristina.lorca@irta.cat (C.L.-O.); rosa.valle@irta.cat (R.V.); nuria.roca@irta.cat (N.R.); albert.bensaid@irta.cat (A.B.); 3 Dipartimento di Virologia, Istituto Zooprofilattico Sperimentale della Lombardia e dell’Emilia Romagna, Via A. Bianchi 9, 25124 Brescia, Italy; santina.grazioli@izsler.it (S.G.); tiziana.trogu@izsler.it (T.T.); anamaria.morenomartin@izsler.it (A.M.); 4 IrsiCaixa AIDS Research Institute, 08916 Badalona, Spain; jcarrillo@irsicaixa.es (J.C.); nizquierdo@irsicaixa.es (N.I.-U.); jblanco@irsicaixa.es (J.B.); mparera@irsicaixa.es (M.P.); mnoguera@irsicaixa.es (M.N.-J.); bclotet@irsicaixa.es (B.C.); 5 IrsiCaixa AIDS Research Institute, Germans Trias i Pujol Research Institute (IGTP), Can Ruti Campus, 08916 Badalona, Spain; 6 Chair of Infectious Diseases and Immunity, Faculty of Medicine, University of Vic-Central University of Catalonia (UVic-UCC), 08500 Vic, Spain; 7 UAB, CReSA (IRTA-UAB), Campus de la UAB, 08193 Cerdanyola del Vallès, Spain; joaquim.segales@irta.cat; 8 Departament de Sanitat i Anatomia Animals, Facultat de Veterinaria, UAB, 08193 Cerdanyola del Vallès, Spain

**Keywords:** SARS-CoV-2, COVID-19, lion, *Panthera leo*, wildlife, zoo

## Abstract

To date, no evidence supports the fact that animals play a role in the epidemiology of the severe acute respiratory syndrome coronavirus 2 (SARS-CoV-2), the causative agent of the coronavirus infectious disease 2019 (COVID-19). However, several animal species are naturally susceptible to SARS-CoV-2 infection. Besides pets (cats, dogs, Syrian hamsters, and ferrets) and farm animals (minks), different zoo animal species have tested positive for SARS-CoV-2 (large felids and non-human primates). After the summer of 2020, a second wave of SARS-CoV-2 infection occurred in Barcelona (Spain), reaching a peak of positive cases in November. During that period, four lions (*Panthera leo*) at the Barcelona Zoo and three caretakers developed respiratory signs and tested positive for the SARS-CoV-2 antigen. Lion infection was monitored for several weeks and nasal, fecal, saliva, and blood samples were taken at different time-points. SARS-CoV-2 RNA was detected in nasal samples from all studied lions and the viral RNA was detected up to two weeks after the initial viral positive test in three out of four animals. The SARS-CoV-2 genome was also detected in the feces of animals at different times. Virus isolation was successful only from respiratory samples of two lions at an early time-point. The four animals developed neutralizing antibodies after the infection that were detectable four months after the initial diagnosis. The partial SARS-CoV-2 genome sequence from one animal caretaker was identical to the sequences obtained from lions. Chronology of the events, the viral dynamics, and the genomic data support human-to-lion transmission as the origin of infection.

## 1. Introduction

Coronavirus infectious disease 2019 (COVID-19), caused by the severe acute respiratory syndrome coronavirus 2 (SARS-CoV-2), is essentially a human disease. However, the potential role of other mammalian species in the epidemiology of the disease either as competent hosts or as potential reservoirs has been in the spotlight [1]. This interest spurred early epidemiological research aiming to predict and identify which animal species could be susceptible to SARS-CoV-2 infection and capable of transmitting the virus to either humans or other animals [2,3]. Experimental infections have attempted to identify potential SARS-CoV-2 hosts as well as animal models to recapitulate the COVID-19 immunopathological features observed in humans [4]. Carnivores and primates have been identified as the most sensitive taxonomic groups to infection, with clinical disease reported in some instances [4]. However, there is still limited information about the role of mammalian species in the epidemiology of COVID-19.

Despite the high incidence of SARS-CoV-2 human infection, the frequency of spillover events to other species has been relatively low [5]. In most instances, cases of infection in non-human mammals have been largely asymptomatic and required extensive efforts to identify them. Pets from COVID-19-affected households have usually shown asymptomatic infections or mild respiratory/digestive clinical signs [6,7,8,9,10]. Other domestic animals have shown less susceptibility to SARS-CoV-2 infection [2,5,11]. In contrast, mink farms proved to be a singular exception with SARS-CoV-2 causing large outbreaks, resulting in massive culling in several countries. Infection in minks also resulted in the only documented transmission of SARS-CoV-2 from non-human mammals to persons [12]. Overall, reports of natural infection and clinical disease in animals have been restricted to isolated cases, most likely identified due to extensive surveillance.

Zoos are singular regarding the epidemiology of human–animal interactions: they house multiple wildlife species of a wide array of taxa in relative proximity and the interactions between animals and humans are frequent, particularly for animal caretakers. The COVID-19 pandemic, therefore, triggered research for data and surveillance for suspicious cases of SARS-CoV-2 infection in zoo animals [13]. Since April 2020, several cases of SARS-CoV-2 infections have been reported in zoo animals [14]. Most reported outbreaks have involved large felids; curiously, only few cases of natural infection have been detected in non-human primates [15].

The first SARS-CoV-2 outbreak identified and reported in a zoo took place at the Bronx Zoo, early in the COVID-19 epidemic in New York City, and resulted in a self-limiting disease affecting tigers (*Panthera tigris*) and lions (*Panthera leo*) [16]. Unlike infections reported in domestic cats, which have been largely asymptomatic, tigers and lions displayed mild to moderate upper respiratory clinical signs (coughing and wheezing), lasting from 1 to 16 days. In addition, animals experienced transitory decreased appetite or anorexia, but all animals recovered spontaneously. Molecular and virological analyses identified the felid caretakers as their primary source of transmission [16,17]. Scarce information has been reported in other large felid infections occurring at zoos in South Africa, the Czech Republic, Sweden, and Sri Lanka [18]. In addition, nine Asiatic lions kept at the Arignar Anna Zoological Park (India) were infected with the SARS-CoV-2 delta variant (pango B.1.617.2 lineage) in May 2021 [19]. The source of infection could not be determined considering none of their caretakers were COVID-19 positive, but the sequenced viruses corresponded to the circulating variants in India at that time [19]. Mild respiratory clinical signs were reported in some animals, while two of the lions died after succumbing to the infection [19]. So far, no virological or pathological data has been reported but fatalities are presumably related to a COVID-19-like disease in these animals. While these studies provide important information on SARS-CoV-2 infection in large felids, many aspects remain unexplored. 

In the present study, we report natural SARS-CoV-2 infection of the four lions at the Barcelona Zoo (Barcelona, Spain) and provide clinical, virological, and immunological data, as well as viral genomic comparison from animals and their caretakers.

## 2. Materials and Methods

### 2.1. Clinical Evaluation and Sample Collection

Animal handling and sampling were performed at the Barcelona Zoo (Catalonia, Spain) as part of the routine management and investigations performed when clinical signs are displayed and potential zoonoses are suspected.

On 8 November 2020, four lions (*Panthera leo bleyenberghi*, three 16-year-old females and one 4-year-old male; L1, L2, L3, and L4, respectively) developed respiratory signs including sneezing, coughing, and nasal discharge. Additionally, the caretakers noticed changes in the behavior and attitude of the animals. Based on a previous report from the Wildlife Conservation Society describing natural infection with SARS-CoV-2 in large felids at the Bronx Zoo and the clinical suspicion for SARS-CoV-2 infection, a RADT was performed to confirm the infection in lions. Additionally, a safety sampling protocol was implemented together with routine animal management monitoring and more precise recording of clinical signs supported by video images captured continually at the indoor lion facilities. In addition, images between the 4 and 23 November were carefully reviewed to determine the potential onset of clinical signs.

Prior to 9 November, lion keepers wore personal protective equipment (PPE) including nitrile gloves and UNE-00065 certified cloth masks when working in the lion facility or preparing their food. From that day onwards, enhanced PPE included donning dedicated whole-body coveralls, FFP3 masks, face shields, and waterproof shoe covers to access the lion facility.

All four lions were target-trained and were compliant to deep nasal swabbing using sterile cotton swabs, which were inserted into the nasal cavity of the subject, briefly rotated, and extracted. Fecal samples were also collected from the facility, despite the fact that the samples could not be linked to a known individual in most instances. Additionally, saliva was obtained from each lion when possible during the week after respiratory clinical signs’ exhibition. All samples were stored at −80 °C and shipped to the IRTA-CReSA facilities (Catalonia, Spain) on cold packs. All samples were collected at different time-points during a 3-week period between 9 November and 2 December 2020, as shown in Supplementary Table S1.

The lions were anesthetized for health examination, including blood extraction and pulmonary ultrasound between 2 and 5 weeks after clinical presentation, and three of them were checked-up again 4 months post-clinical presentation. L1 was not anesthetized 4 months later due to risk assessment; this animal was known for a specific reaction to anesthetic procedures and veterinarians were reticent regarding extra anesthesia. Anesthesia procedures were performed by remote darting following initial premedication with midazolam (0.1–0.12 mg/kg BW) using a combination of ketamine (1.5–2 mg/kg BW), butorphanol (0.1–0.15 mg/kg BW), and detomidine (0.075–0.08 mg/kg BW). Whole-blood samples were obtained by jugular venipuncture using standard procedures into vacuum serum collection tubes (Vacutainer^®^, Beckton Dickinson, San Diego, CA, USA). Blood samples from all lions collected in February 2020 were used as control samples before infection.

### 2.2. Additional Sample Collection

On initial case presentation, additional nasal swabs from other large carnivores held at the Barcelona Zoo were obtained, including hyenas (*Crocutta crocutta*), wolves (*Canis lupus signatus*), bears (*Ursus arctos*), tigers (*Panthera tigris sumatrae*), jaguars (*Panthera onca*), and leopards (*Panthera pardus kotiya*).

Additionally, two lion keepers who were at home on COVID-19 quarantine at the time when the lions showed clinical signs were asked to collect nasal swab self-samples during the week after clinical presentation of the animals. This period corresponded approximately to one week after their confirmation of COVID-19 positivity. The swabs were stored at −20 °C until the quarantine was lifted and the samples were then shipped to the laboratory.

### 2.3. SARS-CoV-2 Antigen Test

Nasal swabs obtained as previously described were tested for the presence of the SARS-CoV-2 nucleoprotein (N) antigen using a commercially available kit (COVID-19 Ag Test reference 243103N-20, nal von minden GmbH) following manufacturer’s instructions. Briefly, nasal swabs were immersed, stirred, and incubated at room temperature for 2 min in 10 drops sample buffer. Then, swabs were removed and 2 drops of the sample solution were transferred to the sample well of the rapid test cassette and incubated for 15 min at room temperature. Results were read by interpreting visible bands at the control line and test line. 

### 2.4. Cell Culture and SARS-CoV-2

Vero E6 cells (ATCC^®^ repository, CRL-1586™) and SARS-CoV-2 stocks (passage 3; hCoV-19/Spain/CT-2020030095/2020; GISAID ID EPI_ISL_510689) were prepared as previously reported in [20,21] in Dulbecco’s modified Eagle Medium, DMEM (Lonza, Basel, Switzerland) supplemented with 5% fetal calf serum (FCS; EuroClone, Milan, Italy), 100 U/mL penicillin, 100 μg/mL streptomycin, and 2 mM glutamine (all from Gibco Life Technologies, Madrid, Spain).

SARS-CoV-2 was isolated from a nasopharyngeal swab collected from an 89-year-old person in Barcelona, Spain, and characterized by genomic sequencing (hCoV-19/Spain/CT-IrsiCaixa-04/2020; GISAID ID EPI_ISL_418268) as previously described [20]. This isolate carries D614G and R682L amino acid substitutions in the spike protein compared to the previously identified Wuhan/Hu-1/2019 reference variant (NCBI accession number: NC_045512.2). The infectious titer of the SARS-CoV-2 stock was calculated by determining the dilution that caused CPE in 50% of the inoculated cell cultures (50% tissue culture infectious dose endpoint, TCID_50_) in Vero E6 cells.

### 2.5. RNA Extraction and Detection by RT-qPCR 

Nasal, saliva, and fecal swabs were transferred into cryotubes containing 500 µL DMEM supplemented with 100 U/mL penicillin, 100 μg/mL streptomycin, and 2 mM glutamine, vortexed and stored at −80 °C until use. Detection of SARS-CoV-2 RNA was performed by a reverse transcriptase-quantitative PCR (RT-qPCR) as previously described [22]. Viral RNA was extracted using the Indimag Pathogen kit (Indical Biosciences, Leipzig, Germany) on a Biosprint 96 workstation (Qiagen, Hilden, Germany) according to the manufacturers’ instructions. Detection of SARS-CoV-2 in RNA extracts was performed following a previously described protocol targeting the E gene (REF), applying minor modifications. Briefly, RT-qPCR was carried out using AgPath-ID^TM^ One-Step RT-PCR Reagents (Applied Biosystems, Life Technologies), and amplification was done by using a 7500 Fast Real-Time PCR System (Applied Biosystems, Life Technologies, Waltham, MA, USA) with the following thermal profile: 10 min at 50 °C; 10 s at 95 °C; and 45 cycles of 15 s at 94 °C and 30 s at 58 °C. Samples with a Cq value ≤ 40 were considered positive for SARS-CoV-2 RNA.

### 2.6. SARS-CoV-2 Genome Sequencing and Phylogenetic Analyses

Viral RNA was extracted as described above and converted to cDNA with the PrimeScript™ RT reagent kit (Takara Bio Europe SAS, Saint-Germain-en-Laye, France) using a combination of oligo-dT and random hexamer methods according to the manufacturer’s protocol. cDNA was used for viral DNA enrichment using the ARTIC-Cov v3 PCR protocol and primer design with the Q5 Hot-start HF polymerase. Resulting PCR products were used for sequencing-ready library preparation with the Illumina DNA LibPrep kit (Illumina, San Diego, CA, USA). Sequencing-ready libraries were then loaded onto the Illumina MiSeq platform and a 150 bp paired-end sequencing kit (300 cycles). Sequence reads were quality-filtered and adapter primer sequences were trimmed using Trimmomatic. Sequencing reads were then mapped against the reference Wuhan/Hu-1/2019 variant (NCBI accession number: NC_045512.2) using the bowtie2 tool [23]. Consensus genomic sequence were generated from the resulting alignments at average depth of coverages ranging from 85 to 1475x using iVar/samtools [24,25]. Genomic sequences from lion specimens were deposited at the GISAID repository with correlative accession IDs from EPI_ISL_1443170 to EPI_ISL_1443186, and EPI_ISL_1740499 for the lion caretaker.

To compare the sequenced genomes obtained from the lion respiratory tract and fecal specimens, as well as the caretaker nasopharyngeal samples, evolutionary analysis was inferred by the maximum likelihood method and the Hasegawa–Kishino–Yano model [26]. The percentage of trees in which the associated taxa clustered together is shown next to the branches. Initial tree(s) for the heuristic search were obtained automatically by applying neighbor-joining and BioNJ algorithms to a matrix of pairwise distances estimated using the maximum composite likelihood approach and then by selecting the topology with the superior log likelihood value. The tree is drawn to scale with branch lengths measured in the number of substitutions per site. The analysis involved 20 nucleotide sequences. Codon positions included were 1st + 2nd + 3rd + Non-coding. All positions with less than 80% site coverage were eliminated, i.e., fewer than 20% alignment gaps, missing data, and ambiguous bases were allowed at any position (partial deletion option). There was a total of 25,132 positions in the final dataset. Evolutionary analyses were conducted in MEGA X [27].

### 2.7. Virus Isolation in Vero E6 Cells

Nasal and fecal swabs positive for SARS-CoV-2 as determined by RT-qPCR were evaluated for the presence of the infectious virus by titration in Vero E6 cells, as previously reported [21,28]. Direct samples and ten-fold dilutions were transferred to Vero E6 monolayers. Plates were monitored daily under light microscope and wells were assessed for the presence of cytopathic effects for 6 days. The amount of infectious virus in the swabs was calculated by determining the TCID_50_.

### 2.8. SARS-CoV-2 RBD and N-ELISA 

Serological assays were performed to detect antibodies against the S and N proteins of SARS-CoV-2. Prior to ELISA tests, serum samples were heat inactivated at 56 °C for 30 min. Afterwards, specific nAbs against the RBD of the S protein were determined in serum samples from all collected time-points using the SARS-CoV-2 sVNT (Genescript) following the manufacturer’s instructions. The specific percentage of inhibition for anti-RBD antibodies was calculated after subtracting the background signal obtained for each sample in antigen-free wells and using the mean OD of the negative control samples. Samples with an activity of ≥30% inhibition were considered positive.

In addition, antibodies targeting the N protein were detected using an in-house double-antigen sandwich ELISA (DAS-N-ELISA). The method uses a recombinant N protein antigen coated to the plate and a recombinant N protein conjugated with HRP (N-HRP), expressed in *Escherichia coli* and purified by immobilized metal affinity chromatography. Briefly, 0.2 µg/mL of the unconjugated recombinant SARS-CoV-2 N protein (50 µL per well) were coated onto ELISA microplates. Tested sera were diluted 1/2.5 in diluent buffer in a final volume of 50 µL, added to the coated plate, and incubated for 60 min at room temperature. Negative and positive controls were included in each plate at the same serum dilution. After three washing cycles, 50 µL/well of the HRP-conjugated recombinant SARS-CoV-2 N protein was added and the plate was incubated for another 60 min at room temperature. After washing, TMB substrate (Surmodics) was added and the ELISA microplates were incubated in the dark at room temperature for 20 min. The colorimetric reaction was then stopped by the addition of 2N sulphuric acid and absorbance values were read at 450 nm using an ELISA reader. Results were expressed as a percentage of reactivity compared with the positive control included in each plate (%S/P), calculated as (OD sample − average OD neg control/average OD pos control − average OD neg control) × 100. More details of the development and analytical performance of the in-house DAS-N-ELISA can be found in the Supplementary Materials (Appendix A).

### 2.9. SARS-CoV-2 Neutralization Assay

The seroneutralization assay using replicating SARS-CoV-2 was performed as previously described [21,29]. Serum samples were inactivated for 30 min at 56 °C. Then, sera were first diluted 1:50 and then were 3-fold serially diluted in supplemented DMEM, mixed 1:1 with the SARS-CoV-2 isolate (EPI_ISL_418268), and incubated 1 h at 37 °C. Then, each dilution mixture (in duplicates) was transferred to Vero E6 monolayers containing 100 TCID50 of SARS-CoV-2 per well and were cultured for 3 days at 37 °C and 5% CO_2_. CPE was measured after 3 days using the CellTiter-Glo luminescent cell viability assay (Promega, Madison, WI, USA) according to the manufacturer’s protocol. Luminescence was measured as luminescence units in a Fluoroskan Ascent FL luminometer (ThermoFisher Scientific, Waltham, MA, USA). Serum virus neutralization titer (SNT50) was defined as the reciprocal value of the sample dilution that showed 50% protection of virus growth. Sera with titers ≥ 1/100 were considered positive for SARS-CoV-2 nAbs.

### 2.10. Data Analysis and Statistics

Mean of binding or nAb data were analyzed using the parametric two-way ANOVA test and paired *t*-test for comparison of 2 groups. The normality of each data set was calculated using the Shapiro–Wilk test and *p*-values lower than 0.05 were considered statistically significant. 

Dose–response curves of serum samples were adjusted to a non-linear fit regression model calculated with a normalized logistic curve with variable slope. For data normalization, uninfected cells and untreated virus-infected cells were used as negative and positive control of infection (% Neutralization = (RLUmax − RLUexperimental)/(RLUmax − RLUmin) × 100), respectively. All SNT50 values were determined as the concentration of sera blocking 50% of the SARS-CoV-2-induced CPE in Vero E6 cells and were expressed as reciprocal dilution. All statistical analyses were performed with GraphPad Prism 8.4.3 (GraphPad Software, Inc., San Diego, CA, USA).

## 3. Results

### 3.1. COVID-19 Confirmation in a Lion Caretaker and Main Chronological Events at the Barcelona Zoo

**Figure 1:**
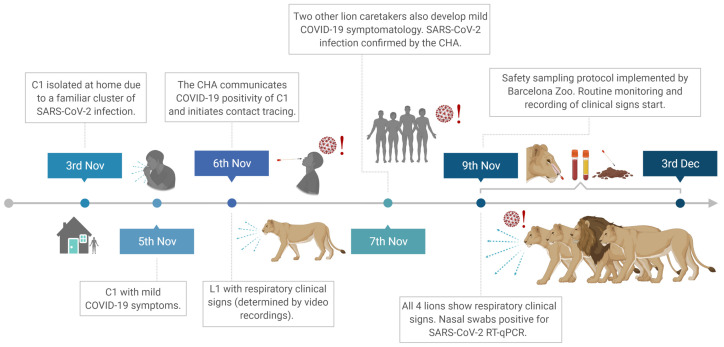
Chronological events summarizing the outbreak and human-to-lion transmission that occurred at the Barcelona Zoo. Abbreviations: C1, lion caretaker 1; COVID-19; coronavirus infectious disease 2019; CHA, Catalan Health Agency. L1, Lion1; SARS-CoV-2, severe acute respiratory syndrome coronavirus 2. This figure was created using BioRender.com.

**Figure 2:**
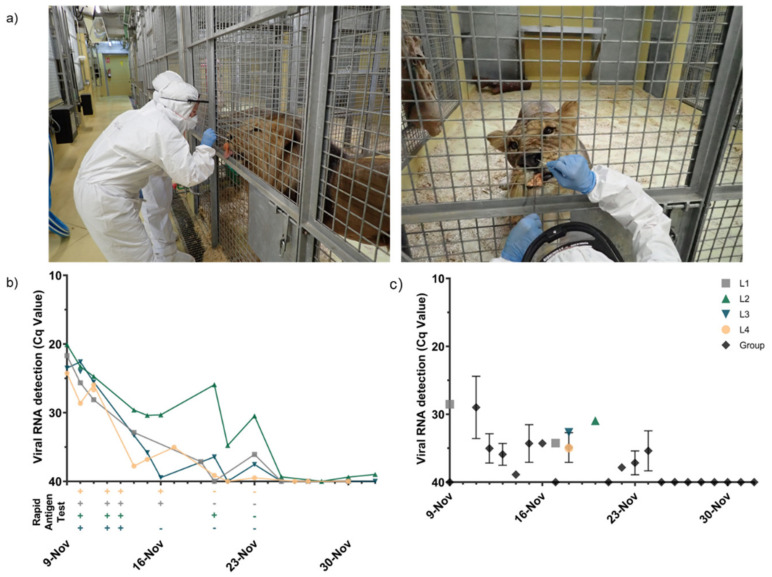
Viral shedding in lions after natural SARS-CoV-2 infection. (**a**) Nasal swab collection procedure in lions. After SARS-CoV-2 infection of the animals was confirmed, trained zoo personnel wore personal protective equipment to access the lion facility and sample the animals using cotton swabs. All four lions were target-trained and were compliant to nasal swabbing by their caretakers. (**b**) Results obtained with a COVID-19 RADT (+, positive; −, negative) and viral RNA levels by RT-qPCR in nasal swab specimens collected from all four lions (L1 to L4). (**c**) SARS-CoV-2 viral RNA levels from individual and collective fecal samples at different time-points after infection. Each colored line/dot represents an individual animal. Black lines/dots indicate collective specimens collected inside the lion facilities. Abbreviations: Cq, quantification cycle; L1, Lion1; L2, Lion2; L3, Lion3; and L4, Lion4.

The main chronological events describing the outbreak and human-to-lion transmission that occurred at the Barcelona Zoo are shown in Figure 1. Briefly, a lion caretaker (C1) was preventively quarantined at home after being in contact with a COVID-19-positive relative. Two days later, C1 developed mild COVID-19 symptomatology, mainly headache. SARS-CoV-2 infection of C1 was confirmed on 6 November 2020 by the Catalan Public Health Agency (CHA) and contact tracing was initiated. During the same night, one lion (L1) displayed respiratory clinical signs (bouts of sneezing and coughing) as determined by video recordings. The next day (November 7th), three caretakers from C1’s team, two of whom attended to the lions from November 4th to 6th (hereafter referred to as C2 and C3), reported mild respiratory symptoms (sore throat, coughing, and fever). All three caretakers tested positive for SARS-CoV-2 as reported by the CHA with a rapid antigen diagnostic test (RADT). On November 9th, caretakers reported all four lions (L1–L4) showing respiratory clinical signs (sneezing, coughing, nasal discharge, and mild apathy). On that day, nasal swab samples were obtained from all lions as shown in Figure 2a. Animal samples were thoroughly tested by RADT and by a specific RT-qPCR to detect viral RNA which reveal to be positive. From this moment onwards, a safety sampling protocol of the animals was implemented by the zoo staff. Lions were observed several times per day through video recording, evaluated for clinical signs, and SARS-CoV-2 infection was monitored daily for 3 weeks. 

To identify and curtail potential transmission of SARS-CoV-2 to humans or other potential susceptible species, the zoo personnel were further screened for SARS-CoV-2 infection during the following days using RADT and/or RT-qPCR. All staff tested, including those exposed to the lions from 5 to 8 November, were negative. Nasal swabs obtained from the other large carnivores (hyenas, wolves, bears, tigers, jaguars, and leopards) held at the zoo were also negative by SARS-CoV-2 RT-qPCR. There were no further suspected or confirmed cases of SARS-CoV-2 among the animal care staff or the animals at the zoo. 

### 3.2. SARS-CoV-2 Antigen, RNA Detection, and Infectious Virus Isolation

Viral RNA was detected in the nasal cavity of the infected lions for two weeks (Figure 2b). Although results showed that the peak of infection already occurred when the sampling started, the amount of SARS-CoV-2 RNA in nasal swabs was still high in all animals when the infection was firstly confirmed (Cq < 25) (Figure 2b). All lions initially had similar levels of SARS-CoV-2 RNA (Cq 20–25), which decreased subsequently over the following 15 days (Figure 2b).

The RADT for the detection of the SARS-CoV-2 N protein remained positive during the first week after the onset of the clinical signs and correlated with the highest viral loads detected by RT-qPCR (Figure 2b). Over the second week, the RADT became negative on the nasal swabs for all four lions, coinciding with a drop in viral RNA load (Figure 2b). SARS-CoV-2 RNA was also detected in saliva samples of all the lions, collected between 13 and 16 November (data not shown). Viral RNA was detected in individual and group feces collected during the study, although at lower levels to those quantified in nasal swabs regardless of the sampled day (Figure 2c). SARS-CoV-2 RNA loads in feces remained low (Cq < 30) from 12 November and onwards (6 days after clinical signs onset in L1), and viral RNA could not be detected 2 weeks later (Figure 2c). From 25 November until the end of the sampling (December 2nd), the nasal swabs and feces of all lions were negative by SARS-CoV-2 RT-qPCR.

RT-qPCR positive nasal and fecal samples with a Cq value < 30 (*n* = 18) were tested for the presence of infectious virus. Infectious SARS-CoV-2 could be determined in two of the samples (L4 and L2 nasal swabs collected on November 9th and 10th, respectively). Direct and 1/10 diluted nasal swab samples produced a cytopathic effect (CPE) on Vero E6 cell cultures. Although viral RNA was detected in fecal samples, isolation of infectious virus was not successful.

### 3.3. SARS-CoV-2 Sequencing Confirmed Human-to-Lion Transmission and Genomic Stability in the New Host

SARS-CoV-2 genomic RNA was sequenced from all lion samples containing Cq values < 30 (*n* = 25; see Figure 2b,c). Among them, 17 high-quality genomic sequences with an optimal coverage depth were obtained (GISAID uploads from EPI_ISL_1443170 to EPI_ISL_1443186). Additionally, partial genomic sequences could also be obtained from a single nasal self-sample of C2 (Cq = 36.61).

**Figure 3:**
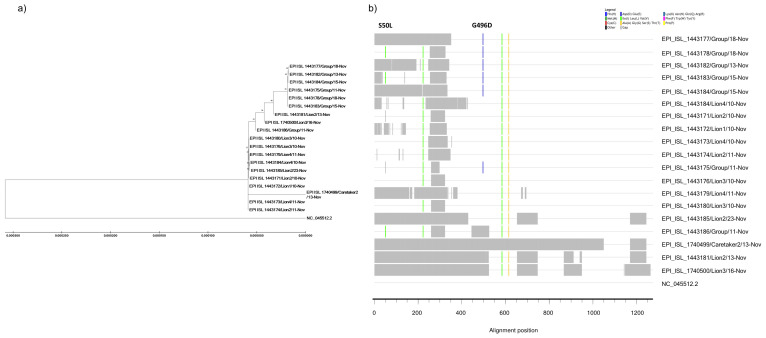
Whole-SARS-CoV-2 genome sequencing and phylogenetic and comparative analyses. (**a**) Maximum likelihood phylogenetic analysis of the genomic sequences obtained from lion respiratory and fecal specimens at different time-points after infection was detected, as well as a nasopharyngeal sample from a lion caretaker, using the Hasegawa–Kishino–Yano model. The reference SARS-CoV-2/Wuhan-Hu-1 isolate (NCBI accession number: NC_045512.2) was included as an outgroup of the analysis. The tree with the highest log likelihood (−34294.32) is shown. The percentage of trees in which the associated taxa clustered together is shown next to the branches. The phylogenetic tree is drawn to scale, with branch lengths measured in the number of substitutions per site. (**b**) Amino acid comparative sequence analysis of the spike protein of SARS-CoV-2. The panel shows mismatches (gray boxes) of the sequences obtained from lion respiratory and fecal specimens obtained throughout the study, as well as its respective caretaker, compared to the reference NC_045512.2 sequence. Comparative analyses determined the emerging mutations S50L and G496D specifically in fecal samples, whose positions have been pointed out in the plot. Colored lines in each position of the alignment show amino acid differences and sequencing gaps found among the different analyzed samples according to the legend. Abbreviations: C2, lion caretaker2; L1, Lion1; L2, Lion2; L3, Lion3; and L4, Lion4.

Whole-viral genome sequencing showed that lions were infected with a unique variant of SARS-CoV-2. The SARS-CoV-2 variant of lions belonged to the pango lineage B.1.177. This lineage carries the A222V, D614G, and E583V mutations in the spike (S) protein in comparison to the reference SARS-CoV-2/Wuhan-Hu-1 isolate (NCBI accession number: NC_045512.2). SARS-CoV-2 sequences obtained from the nasal samples from all lions had 100% identity among them and with the partial sequences obtained from C2 (Figure 3a). Moreover, continued sampling and sequencing of SARS-CoV-2 for three weeks revealed that no adaptations to the respiratory tract of the lions occurred. This was especially evident in L2 samples for which high-quality sequencing data was repeatedly obtained for two weeks (coverage depths ranging from 695 to 1031x) and no amino acid mutations in the S region were identified (Figure 3a,b; GISAID IDs EPI_ISL_1443171, EPI_ISL_1443174, EPI_ISL_1443181, and EPI_ISL_1443185). By contrast, all fecal samples analyzed contained two mutations in the viral genome. Curiously, both mutations detected in fecal samples occurred in the S protein. Comparative analysis of the S protein revealed that these mutations resulted in a substitution of serine for leucine and glycine for aspartic acid at amino acid positions 50 (S50L) and 496 (G496D), respectively (see Figure 3b). These specific mutations were investigated in SARS-CoV-2 genomic public databases and were not found in the main variants circulating since the beginning of the pandemic. Among more than 2.2 million SARS-CoV-2 genomic sequences available, the spike S50L mutation has been identified only in 266 genomic sequences, being detected in China early in February 2020 and later in five continents around the globe. Interestingly, when assessing for the presence of this mutation in SARS-related coronaviruses (SARSr-CoVs), S50L was also found in samples from Malayan pangolin and the *Rhinolophus* bat species. Conversely, the spike G496D mutation was only detected in four human infections but also in fecal specimens from *Rhinolophus* bats containing SARSr-CoVs (EPI_ISL_412976, EPI_ISL_412977, EPI_ISL_1098866, EPI_ISL_1699443, EPI_ISL_1699446, EPI_ISL_1699447, and EPI_ISL_1699449). 

### 3.4. Kinetics of Humoral Immune Responses Elicited after SARS-CoV-2 Infection

**Figure 4:**
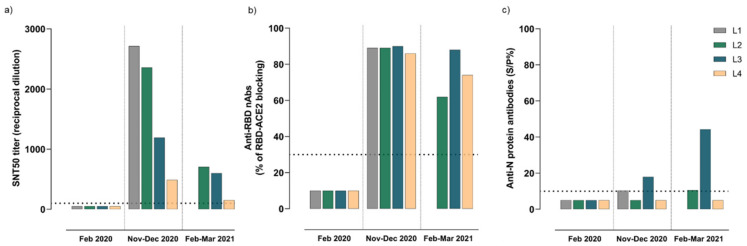
Antibody responses to SARS-CoV-2 elicited in sera from naturally infected lions (L1-L4). (**a**) SARS-CoV-2 neutralizing antibodies determined by the seroneutralization assay. (**b**) Virus-neutralizing antibodies targeting the RBD of the SARS-CoV-2, as determined by the surrogate virus neutralization assay. (**c**) Reactive antibodies against the nucleocapsid (N) protein of the SARS-CoV-2 measured by an in-house ELISA. Each colored bar represents an individual animal. The horizontal dotted lines indicate the cutoff of each assay. Abbreviations: SNT50, serum virus neutralization titer conferring the reciprocal value of the sample dilution that showed 50% protection of virus growth; RBD, receptor-binding domain; nAbs, virus neutralizing antibodies; ACE2, angiotensin-converting enzyme 2; L1, Lion1; L2, Lion2; L3, Lion3; L4, Lion4; N, nucleocapsid protein; and S/P%, percentage of reactivity compared with the positive control included in each assay.

Specific antibody responses targeting SARS-CoV-2 were evaluated in sera samples collected before and after SARS-CoV-2 infection of the lions. None of the animals had antibodies against SARS-CoV-2 prior to infection. Seroconversion was confirmed at 2 to 5 weeks after displaying respiratory clinical signs, as determined by the serum neutralization test (SNT) and surrogate virus neutralization technique (sVNT) (Figure 4a,b, respectively). All lions developed high levels of neutralizing antibodies (nAbs) after infection (Figure 4a), some of which specifically targeted the receptor-binding domain (RBD), as shown in Figure 4b. Although not statistically significant, nAbs and those antibodies targeting the RBD decreased over a 4-month period after infection (Figure 4a,b, respectively). Seroneutralization curves adjusted to a non-linear fit regression model and determination of SNT50 titers are shown in Supplementary Figure S1. In contrast, a limited antibody response against the N protein was found in L3, which increased 4 months after SARS-CoV-2 infection (Figure 4c). Response to the N protein was low in L1 and L2, close to the detection limit, and it was negative in L4 at both time-points after infection (Figure 4c). 

## 4. Discussion

In the present study we reported extensive clinical, virological, and immunological insights of natural SARS-CoV-2 infection in lions. The investigated outbreak occurring in four lions from the Barcelona Zoo is consistent with previous reports of non-domestic large felids being infected with SARS-CoV-2 (*Panthera tigris*, *Panthera leo*, and *Puma concolor*) [16,17,18,19,30]. The chronological events and further genomic characterization fully support human-to-animal transmission of SARS-CoV-2. Moreover, viral genomic sequences retrieved from the respiratory specimens of the lions and their direct keepers were identical, confirming SARS-CoV-2 transmission. Although the transmission route could not be determined (lion keepers wore cloth facial masks inside the lion facility or when preparing their food), the main hypothesis points to close contact transmission between lions and their caretakers; however, human-to-animal indirect transmission or potential transmission events among lions cannot be excluded.

After displaying mild respiratory clinical signs (sneezing, coughing, nasal discharge, and mild apathy), all four lions were rapidly confirmed as infected by SARS-CoV-2. In fact, infection was monitored for a 3-week period at both RNA and protein levels, as determined by RT-qPCR and RADT, respectively, recognizing the N protein of SARS-CoV-2. Although diagnostic sensitivity of RADT is limited, it appears to be a useful tool to monitor SARS-CoV-2 infection in zoo animals. The animals shed viral RNA for approximately 2 weeks after the onset of clinical signs and confirmation of the viral infection. Although the peak of infection occurred slightly before the first sampling and viral shedding varied among individuals, infectious SARS-CoV-2 could be isolated from the nasal cavity of two out of four animals. Additionally, viral RNA was determined in other specimens such as saliva and feces, the latter containing limited loads of viral RNA and no infectious virus could be cultured from any of the collected fecal samples during the study. Although fecal sampling has been proposed as a valid surrogate to monitor infection in these large carnivores, it may be less sensitive than respiratory sampling and may not parallel the described respiratory disease. This is especially relevant for lion management and safe sample collection at zoo facilities. 

Furthermore, whole-viral genome sequencing showed that all lions were infected with the same virus. The SARS-CoV-2 variant infecting lions belonged to the pango lineage B.1.177, which indeed was circulating in Europe (including Barcelona, Spain) in November 2020 when the human-to-lion transmission occurred. This lineage contains A222V, E583V, and D614G mutations in the S protein, which altered SARS-CoV-2 fitness and were fixed in the human population during 2020, with an increased transmissibility compared to other viral lineages [22,31,32]. Distinct viral variants have also infected large felids in different zoos worldwide [17,19,30], which can potentially create new niches for SARS-CoV-2 natural reservoirs. More studies are required to assess the impact of these mutations towards a potential broadening of the susceptible host range as already observed with other SARS-CoV-2 variants [33]. 

Importantly, the infection of lions resulted in high-genomic stability and no amino acid mutations were found in high-quality genomic sequences from respiratory specimens monitored for up to 2 weeks. However, we identified two punctual mutations (S50L and G496D) in the genomes of all fecal samples. S50L mutation specifically occurred in the N-terminal domain of the SARS-CoV-2 S protein, while G496D is found in the receptor-binding motif of the RBD domain. These mutations have also spontaneously emerged in infected humans around the globe but at a low prevalence and it has not been established within the currently circulating SARS-CoV-2 variants. Notably, both S50L and G496D mutations identified in feces from naturally SARS-CoV-2-infected lions were also found in fecal samples from different *Rhinolophus* bat species (*R. affinis*, *R. malayanus*, *R. shameli*, *R. sinicus*, *R. stheno*, *R. pusillus*, and other several *Rhinolophus* species) sampled during the 2010–2020 period in different geographical locations such as the Chinese province of Yunnan as well as Cambodia [34]. Curiously, the closest SARSr-CoV known to date (RaTG13/2013), which shares 96.1% homology to SARS-CoV-2, as well as other SARSr-CoVs characterized in Malayan Pangolins sampled during 2017–2019 [35], also possesses a leucine in the amino acid position 50 of the S protein. In addition, an aspartic acid at position 496 in the S protein (D496) has been continuously found in SARSr-CoVs containing the L50 residue recovered from bat feces sampled during 2018–2019 [34]. Worthy to note, the viral evolution results presented here are in line with the only previous study providing genomic SARS-CoV-2 sequences from naturally infected large felids [17,19]. McAloose et al. sequenced the SARS-CoV-2 genomes from respiratory specimens of a naturally infected tiger at the Bronx Zoo, along with others from the fecal samples of four tigers and three lions. In addition, Mishra and collaborators sequenced the viruses from nasal swabs of four lions. Interestingly, none of these mutations were found in SARS-CoV-2 sequences obtained from the respiratory tract of tigers and lions [17,19], which also showed high-viral genomic stability and coincided with our studies performed in the upper respiratory tract. However, the G496D mutation was present in the SARS-CoV-2 sequences also obtained from feces of three naturally infected tigers at the Bronx Zoo [17]. The presence of the G496D mutation exclusively in the two reported outbreaks providing SARS-CoV-2 sequences in feces from large felids might not be just incidental. Further studies are needed to elucidate the capabilities of SARS-CoV-2 to replicate in different anatomic compartments of large feline species and whether they impose different bottleneck pressures to the virus replicating inside the same host. 

Our results also showed that naturally infected lions cleared the infection and recovered from respiratory clinical signs 2–3 weeks after infection, developing robust humoral responses against SARS-CoV-2 already at 2–5 weeks after infection. In line with previous studies in one tiger [17], all lions developed high titers of nAbs against the S protein and specifically the RBD domain of SARS-CoV-2. Nonetheless, as similarly reported in domestic felines [2,6,36,37], limited levels of antibodies against the nucleoprotein were observed in three of the animals. Moreover, the levels of nAbs decreased four months after infection in all analyzed lions. Due to the lack of specific reagents, it was not possible to determine the isotype of the reactive antibodies against SARS-CoV-2, although IgGs are probably the most abundant ones in serum. More studies are needed to determine the duration of immunity developed by the lions and whether the animals develop sterilizing immunity or, conversely, they could be susceptible to re-infections.

In summary, SARS-CoV-2 infection dynamics in large felids from the Barcelona Zoo were monitored in respiratory and fecal samples for a period of 4 months. The viral variant belonging to the B.1.177 lineage was confirmed to jump from humans to lions and caused respiratory disease in both species without the need of specific mutations. Lions cleared viral infection within three weeks and developed high levels of neutralizing responses to SARS-CoV-2 that lasted at least for four months.

## Data Availability

All data is available upon request to the authors.

## Supplementary Materials

The following is available online at https://www.mdpi.com/article/10.3390/v13091683/s1: Figure S1. Seroneutralization curves adjusted to a non-linear fit regression model and SNT50 titers; Table S1. Number and type of specimens chronologically collected for virology analyses after lions displayed respiratory clinical signs.

## Appendix A

Development and analytical performance of the in-house DAS-N-ELISA.

The positivity threshold, established at 10%, was set for human sera by analyzing 473 samples collected from SARS-CoV-2-positive and negative individuals. Of these, 379 serum samples were from SARS-CoV-2-negative individuals collected in 2012–2016 and 94 were collected from patients for whom the diagnosis of SARS-CoV-2 was made by molecular and/or antigenic or serological analyses. Analytical specificity was also assessed by using rabbits and guinea pigs’ immune sera produced against three different Beta coronaviruses (Bovine Beta-CoV 9WBL77 strain, Porcine hemagglutinating encephalomyelitis virus (PHEV) ATCC VR-741, and Human CoV OC43 ATCC VR-1558). For each virus, six animals (three rabbits and three guinea pigs) were inoculated subcutaneously with partially purified inactivated antigen in complete Freund’s adjuvant and boosted via the same route with the same antigen once in an interval of 21–30 days. Their positive serological reactivity with the homologous antigens was demonstrated by VNT. Two-fold serial dilutions (starting from ¼) of the experimental sera were incubated with 100 TCID50 of the homologous virus. Serum virus neutralization titer (NT) was defined as the reciprocal value of the sample dilution that showed 100% protection of virus growth. NTs for OC43-positive rabbit and guinea pig sera were between 1/128 and 1/256, and 1/28 and 1/256, respectively. Bov-CoV-positive sera showed NTs with the homologous virus between 1/128 and 1/256 for rabbit sera and 1/128 for guinea pig sera. NTs of HEV-positive rabbit and guinea pig sera were 1/256 and between 1/128 and 1/256, respectively.

Polyclonal immune sera were obtained in compliance with the national legislation upon authorization by the competent authority (Italian Ministry of Health D.lgs 26/2014, authorization number 307/2020-PR). All these polyclonal sera were negative with the DAS-N-ELISA, demonstrating the absence of cross-reactions with other Beta-CoVs.
