# Supplementary material for: Monitoring Natural SARS-CoV-2 Infection in Lions (Panthera leo) at the Barcelona Zoo: Viral Dynamics and Host Responses
Source: Viruses. 2021 Aug 25;13(9):1683. doi: 10.3390/v13091683 (PMC8472846; doi:10.3390/v13091683)
Supplement: Supplementary file 1 [file viruses-13-01683-s001.zip › viruses-1357655-supplementary.pdf]

Table S1. Number and type of specimens chronologically collected for virology analyses after lions displayed respiratory clinical signs. L1, Lion1; L2, Lion2; L3, Lion3; L4, Lion4.

	Nasal Swab				Saliva				Feces				
**Date of collection**	**L1**	**L2**	**L3**	**L4**	**L1**	**L2**	**L3**	**L4**	**L1**	**L2**	**L3**	**L4**	**Group**
09-nov	1	1	1	1					1				2
10-nov	1	1	2	1									
11-nov	1	1	1	2									3
12-nov													2
13-nov					1	1		1					5
14-nov	1	1	1	1									2
15-nov		1	1	1									6
16-nov		1	1				1						1
17-nov				1					1				1
18-nov											1	1	5
19-nov	1												
20-nov	1	1	1	1						1			
21-nov		1	1	1									2
22-nov													1
23-nov	1	1	1	1									5
24-nov													5
25-nov	1	1	1										5
26-nov				1									3
27-nov	1		1	1									3
28-nov		1											4
29-nov													2
30-nov	1	1	1	1									3
01-dic													4
02-dic		1	1										1

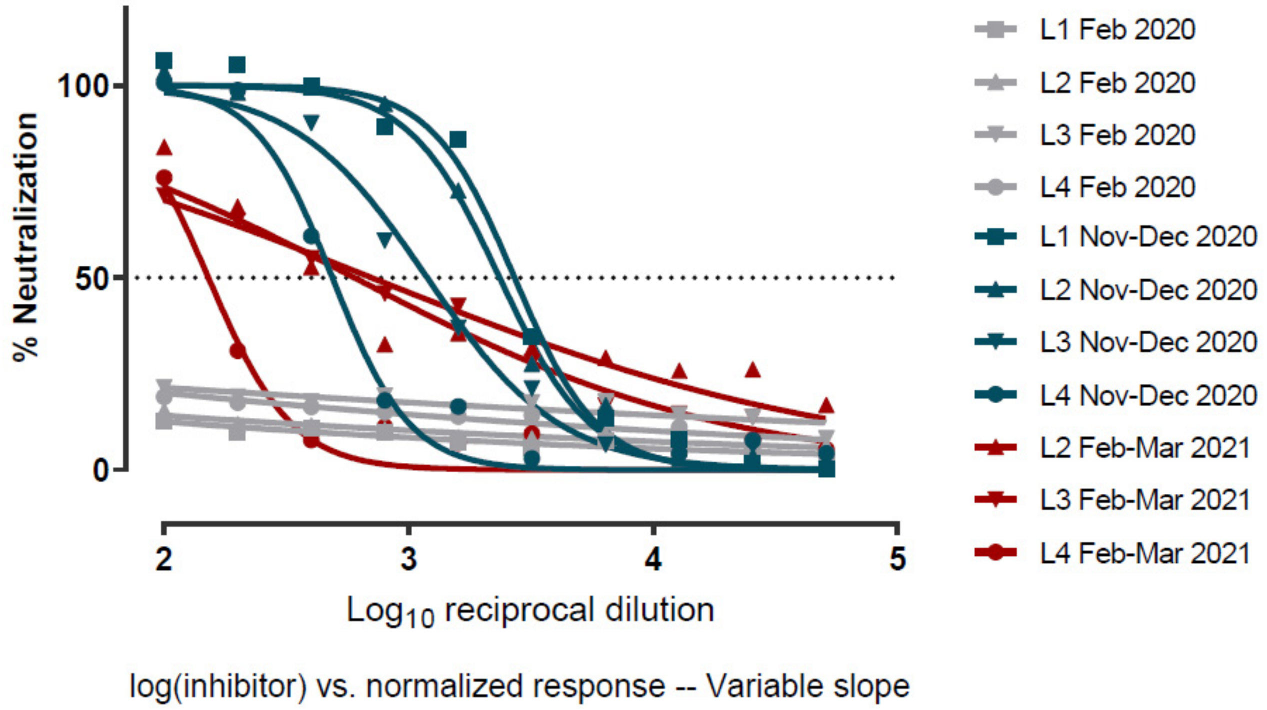

Figure S1. Seroneutralization curves adjusted to a non-linear fit regression model and SNT50 titers.
